# Heterochromatin heterogeneity in *Hypostomus* prope *unae* (Steindachner, 1878) (Siluriformes, Loricariidae)from Northeastern Brazil

**DOI:** 10.3897/CompCytogen.v5i4.1149

**Published:** 2011-11-09

**Authors:** J.A. Bitencourt, P.R.A.M. Affonso, L. Giuliano-Caetano, A.L. Dias

**Affiliations:** 1 Departamento de Biologia Geral, Universidade Estadual de Londrina, CCB, Londrina - 86051-970, Paraná, Brazil; 2 Departamento de Ciências Biológicas, Universidade Estadual do Sudoeste da Bahia, DCB, Jequié - 45200-000, Bahia, Brazil

**Keywords:** C-banding, heterochromatin, ichthyofauna, restriction enzymes

## Abstract

Cytogenetic analyses using C-banding and chromosomal digestion by several restriction enzymes were carried out in four populations (named A, B, C and D) of *Hypostomus* prope *unae* (Loricariidae, Hypostominae) from Contas river basin, northeastern Brazil. These populations share 2n=76 and single NORs on the second metacentric pair but exclusive karyotype forms for each locality. Populations A and B presented conspicuous terminal and interstitial heterochromatic blocks on most of acrocentric chromosomes and equivalent to NORs with differences in both position and bearing pair. Population D showed evident marks at interstitial regions and interspersed with nucleolar region while population C presented interstitial and terminal heterochromatin segments, non-coincident with NORs. The banding pattern after digestion with the endonucleases *Alu* I, *Bam* HI, *Hae* III and *Dde* I revealed a remarkable heterogeneity within heterochromatin, allowing the identification of distinctive clusters of repeated DNA in the studied populations, besides specific patterns along euchromatic regions. The analysis using restriction enzymes has proved to be highly informative, characterizing population differences and peculiarities in the genome organization of *Hypostomus* prope *unae*.

## Introduction

Restriction enzymes (RE) represent a powerful tool for studies about DNA organization (Lima-de-Faria et al. 1980). Such bacterial endonucleases recognize and cleavage target-sequences in the double-strand DNA, providing a highly specific pattern of chromosomal banding according to each enzyme (Lloyd and Thorgaard 1988). The removal of DNA fragments allows studying both structure and base composition of specific chromosomal regions (Lorite et al. 1999; Sánchez et al. 1990, 1991; Lozano et al. 1991; Bianchi et al. 1985). Therefore, the RE banding pattern is an exceptionally sensitive method in heterochromatin analysis (Pieczarka et al. 1996), being able to reveal a higher degree of heterogeneity and more refine comparative analyses than the traditional C-banding itself.

In spite of the intensive application of restriction enzymes in chromosomal analyses of several animal groups (Miller et al. 1976; Kaelbling et al. 1984; Lima-de-Faria et al. 1980; Bianchi et al. 1985; Marchi and Mezzanotte 1988, 1990; Juan et al. 1990; Pieczarka et al. 1996), a few studies of RE-based heterochromatin differentiation are reported in fish chromosomes, being restricted to some groups such as Characidae (Kantek et al. 2007; Maistro et al. 1999), Prochilodontidae (Maistro et al. 2000), Pimelodidae (Swarça et al. 2005; Carvalho and Dias 2005), Salmonidae (Lloyd and Thorgaard 1988; Sánchez et al. 1990, 1991; Lozano et al. 1991; Albuín et al. 1994), Muraenidae (Cau et al. 1988) and Scophthalmidae (Bouza et al. 1994).

Within the genus *Hypostomus* Lacépède,1803, heterochromatin can be associated to heteromorphic chromosomes (Cereali et al. 2008; Kavalco et al. 2004, 2005), sex chromosomes (Artoni et al. 1998) and polymorphism cases (Rubert et al. 2008). In addition, species of this genus usually present a remarkable variability in both distribution and composition of heterochromatin (Artoni and Bertollo 1999). However, these data refer to C-banding and/or fluorochrome staining while studies using enzymatic digestion have not been reported in the genus or the family Loricariidae so far.

The goal of the present work was to analyze comparatively metaphase chromosomes of *Hypostomus* prope *unae* (Steindachner, 1878) by C-banding and RE digestion in order to refine previous cytogenetic studies (Bitencourt 2010) among four populations of this species along a poorly studied coastal river basin in northeastern Brazil.

## Methods

**Figure 1.**
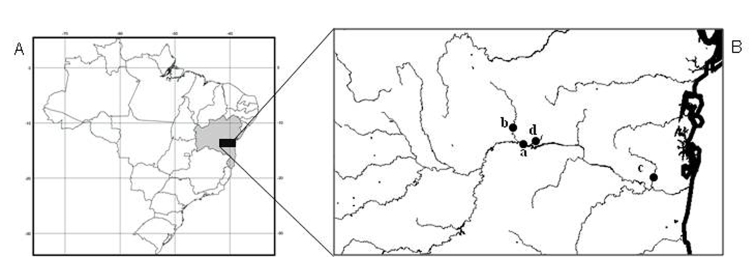
**A–B** Collection sites **A** Map of Brazil, highlighting the state of Bahia in northeastern region; **B** Contas river basin and respective sampling sites: **a**- Contas river, **b**- Preto do Costa river, **c**- Oricó river, **d**- Preto do Criciúma river.

Forty-six specimens of *Hypostomus* prope *unae* from four collection sites in Contas river basin were analyzed, being 10 (3 males, 2 females and 5 immature) from the main channel of Contas river (13°51'51"S, 40°04'54"W), 10 (6 males, 1 female and 3 immature) from Preto do Costa river (13°45'84"S, 39°56'47"W), 15 (9 males and 6 immature) from Oricó river (14°08'03"S, 39°21'30"W), and 11 (4 males, 4 females and 3 immature) from Preto do Criciúma river (13°55'45"S, 39°57'57"W) (Fig. 1).

Voucher specimens were identified by Dr. Claudio Zawadski from Universidade Estadual de Maringá (UEM) and deposited in the fish collection at NUPELIA – UEM, Maringá, PR, Brazil (NUP 9811, 9814). These four populations are referred as A, B, C and D, respectively.

Metaphase chromosomes were obtained from kidney cells as described by Bertollo et al. (1978) after mitotic stimulation using yeast suspension (Lee and Elder 1980) or, alternatively, Munolan® (bacterial and fungal antigens) diluted in water (1 pill per 0.5mL of water), as suggested by Molina (2001). The chromosomes were classified into metacentric (m), submetacentric (sm), subtelocentric (st) and acrocentric (a), as commonly described in fish (Levan et al. 1964). The fundamental number (FN) was established taking into account that m, sm and st chromosomes are bi-armed while chromosomes bear one chromosomal arm.

C-positive heterochromatin was detected according to Sumner (1972), with slight modifications. *In situ* digestion using restriction enzymes was performed as proposed by Mezzanotte et al. (1983), with modifications. Concentration and incubation tests were extensively performed to optimize the results. After defining the best concentration (Table 1), we added 30 μl of each enzyme solution (diluted in specific buffer and distilled water) onto chromosomal preparations. The slides were incubated in moist chamber at 37°C for specific periods according to each enzyme (Table 1). Afterwards, the slides were washed in distilled water and stained with 5% Giemsa in phosphate buffer (pH 6.8) for 8 minutes.

**Table 1.** List of restriction endonucleases (RE) used on the chromosomal preparations of *Hypostomus* prope *unae*, with their respective restriction sites and optimum concentrations and incubation periods obtained in the present work.

**Endonucleases**	**Restriction site**	**Concentration**	**Incubation**
*Alu* I	(5’- AG **↓** CT - 3’)	0.4 U/ µl	4h
*Bam* HI	(5’- G **↓** GATCC - 3’)	0.5 U/ µl	15h
*Hae* III	(5’-GG **↓** CC - 3’)	0.6 U/ µl	14h
*Dde* I	(5’- C **↓** TNAG – 3’)	2 U/ µl	4h

## Results

**Figure 2.**
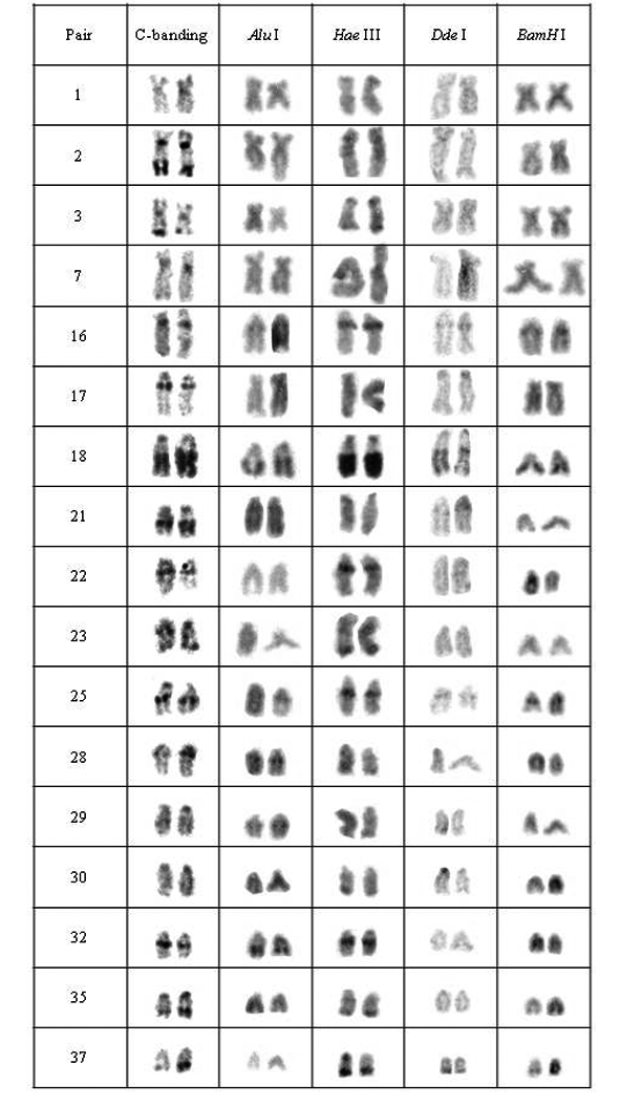
Chromosomal pairs from population A of *Hypostomus* prope *unae* showing the C-positive heterochromatin and banding pattern after digestion with the restriction endonucleases: *Alu* I*, Hae* III, *Dde* I and *Bam* HI.

**Figure 3.**
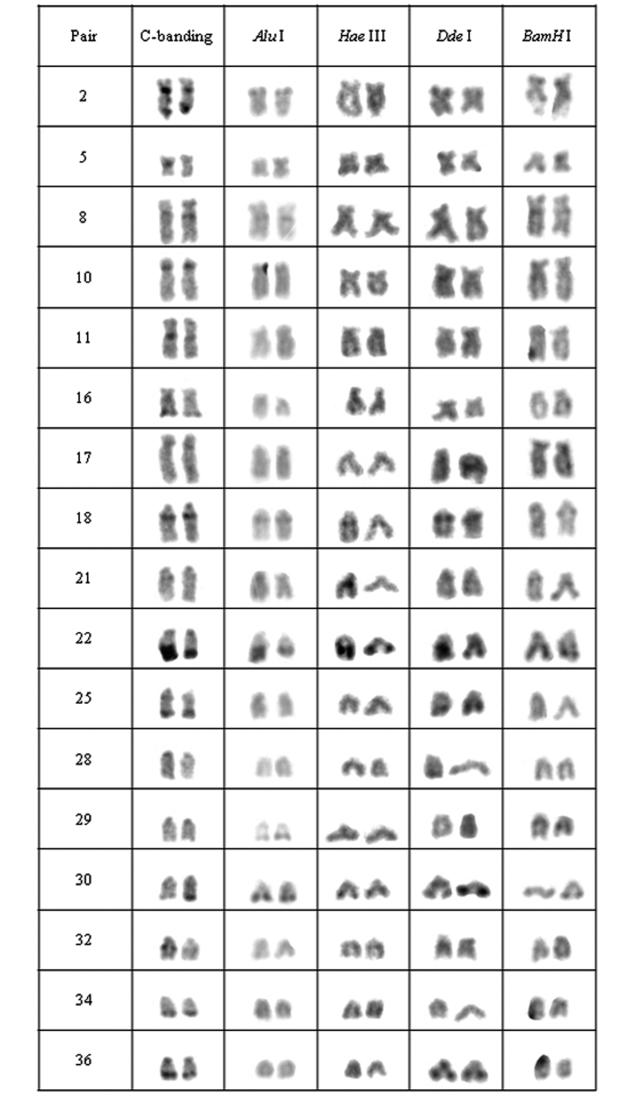
Chromosomal pairs from population B of *Hypostomus* prope *unae* showing the C-positive heterochromatin and banding pattern after digestion with the restriction endonucleases: *Alu* I*, Hae* III, *Dde* I and *Bam* HI.

The specimens from all analyzed populations presented a modal chromosomal number of 2n=76 and distinct karyotype formulae, as follows: 12m+16sm+48st/a (FN= 104) for specimens from population A, 12m+20sm+44st/a (FN=108) for specimens from population B, 10m+14sm+52st/a (FN=100) for individuals from population C and 10m+20sm+46st/a (FN= 106) for those from population D. Furthermore, distinctive patterns of heterochromatin distribution were detected by C-banding. Although populations A and B bear conspicuous terminal and interstitial marks in 17 chromosomal pairs as well as centromeric and NOR-associated heterochromatin, they differ in relation to C-bands position or bearing pair (Figs 2, 3).

**Figure 4.**
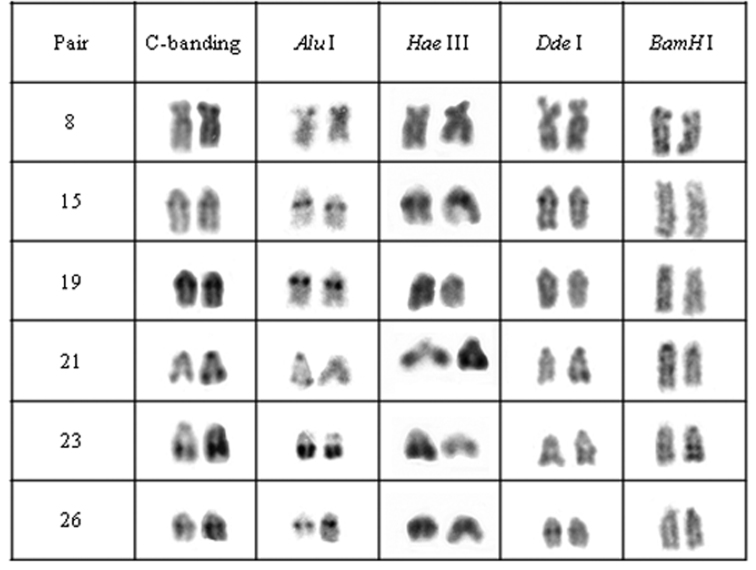
Chromosomal pairs from population C of *Hypostomus* prope *unae* showing the C-positive heterochromatin and banding pattern after digestion with the restriction endonucleases: *Alu* I*, Hae* III, *Dde* I and *Bam* HI.

**Figure 5.**
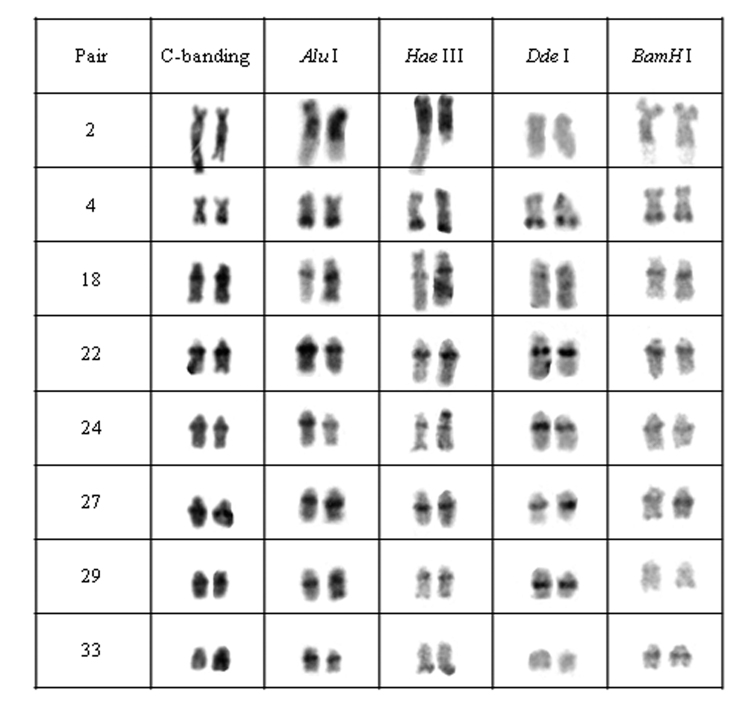
Chromosomal pairs from population D of *Hypostomus* prope *unae* showing the C-positive heterochromatin and banding pattern after digestion with the restriction endonucleases: *Alu* I*, Hae* III, *Dde* I and *Bam* HI.

Heteromorphic blocks were also evident in both populations. Besides the NOR-bearing pair, 18^th^, 21^st^ and 37^th^ pairs in population A and the 22^nd^ pair in population B size differences between homologous (Figs 2, 3). Population C was characterized by interstitial and terminal marks in six chromosomal pairs, non-coincident with NORs (Fig. 4). On the other hand, population D presented eight pairs, most of them acrocentric, bearing interstitial C-bands and also interspersed with NORs (Fig. 5).

The digestion pattern using RE allowed identifying inter-population differences in several chromosomal regions but most in heterochromatin as shown in Table 2, where + stands for digested C-band and – stands for undigested heterochromatic region.

**Table 2.** Heterochromatin digestion pattern using the restriction enzymes *Alu* I*, Hae* III*, Dde* Iand *Bam* HI per population of *Hypostomus* prope *unae*: (+) digested heterochromatin; (-) undigested heterochromatin; (±) partially digested heterochromatin.

**Population**	**C-banded pair.**	**Restriction Enzyme**			
		***Alu*I**	**II*IHa*e**	***IDd*e**	**H*IBa*m**
**A**	1	+	±	+	+
	2	+	+	+	+
	3	+	±	+	+
	7	+	–	+	+
	16	–	–	–	–
	17	+	+	+	+
	18	–	–	–	–
	21	+	+	+	+
	22	+	–	+	+
	23	+	–	+	+
	25	–	–	–	–
	28	–	+	+	+
	29	±	+	+	+
	30	+	+	+	+
	32	+	–	+	+
	35	+	±	+	+
	37	+	–	–	–
**B**	2	+	+	+	+
	5	+	+	+	+
	8	–	+	+	–
	10	+	+	+	+
	11	+	+	+	+
	16	+	+	+	+
	17	+	+	+	+
	18	–	–	–	–
	21	+	+	+	–
	22	–	–	–	–
	25	+	+	–	+
	28	+	+	+	+
	29	+	+	+	+
	30	–	–	–	–
	32	+	–	+	–
	34	+	+	+	+
	36	+	+	–	+
**C**	8	–	+	–	–
	15	–	–	–	+
	19	–	–	+	+
	21	–	–	±	±
	23	–	–	–	±
	26	–	–	–	+
**D**	2	+	+	+	+
	4	–	–	–	–
	18	–	–	–	–
	22	–	–	–	–
	24	–	–	–	–
	27	–	–	–	–
	29	–	–	–	+
	33	–	+	+	–

Five heterochromatin (or repeated DNA) groups were identified in population A: (a) the heterochromatin from pairs 2, 17, 21, 30, centromeric heterochromatin of pairs 1, 3, and 35, and terminal regions of the 29^th^ pair were digested by all tested enzymes; (b) the chromosomal pairs 16, 18 and 25 lacked any target sequences; (c) pairs 7, 22, 23, 32 and the terminal heterochromatin of pairs 1, 3 and 35 were digested by *Alu* I, *Bam* HI and *Dde* I; (d) pair 28 and the upper portion of the heterochromatic block in pair 29 were digested by *Hae* III, *Bam* HI and *Dde* I; and (e) the 37^th^ pair was digested by *Alu* I (Fig. 2).

In population B, the heterochromatin was divided into six groups: (a) the heterochromatin from pairs 2, 5, 10, 11, 16, 17, 28, 29 and 34 were digested by all enzymes; (b) the chromosomal pairs 18, 22 and 30 lacked the target sequences; (c) the pairs 25 and 36 were digested by *Alu* I, *Bam* HI and *Hae* III; (d) the heterochromatin from pair 8 was digested by *Hae* III and *Dde* I; (e) the 32nd pair was digested by *Alu* I and *Dde* I; (f) and the 21^st^ pair was digested by *Alu* I, *Hae* III and *Dde* I (Fig. 3).

Enzymatic digestion of heterochromatic regions in population C revealed four heterochromatin groups: (a) centromeric region of pair 21 and the terminal blocks in pair 23 remained intact; (b) pair 8 was digested by *Hae* III; (c) pair 15, central portion of heterochromatic block in pairs 23 and were digested by *Bam* HI; (d) pair 19 and the terminal region of pair 21 were digested by *Bam* HI and *Dde* I (Fig. 4).

Heterochromatin regions in population D were also divided into four groups: (a) pair 2 was digested by all enzymes; (b) pairs 4, 18, 22, 24 and 27 were not digested by the tested enzymes; (c) the 29^th^ pair presented target sequences for *Bam* HI; (d) and the 33^rd^pair was digested by *Hae* III and *Dde* I (Fig. 5).

Independently on the population, the nucleolus organizer regions (2^nd^ pair) were digested by all restriction enzymes, including those samples in which NOR-associated heterochromatin was not detected by C-banding.

In relation to the digestion pattern in euchromatic regions, some conspicuous bands were observed, being specific for each population and enzyme. In general, population A presented a high number of chromosomes bearing *Hae* III bands, whereas populations B and C presented larger amounts of *Alu* I bands. On the other hand, population D was characterized by a large number of chromosomes bearing bands after treatments with all enzymes (data not shown).

## Discussion

Chromosomal digestion by restriction endonucleases results in a faint chromosomal staining and identification of a characteristic band pattern according to each enzyme (Lima-de-Faria et al. 1980). The decreased chromatin staining is considered a reliable evidence of the removal of DNA fragments by RE once Giemsa attaches to DNA directly (Miller et al. 1983; Bianchi et al. 1985; Kaelbling et al. 1984), but other factors might also play an important role in this pattern.

A hindered access to chromosomal DNA has been pointed out as an alternative explanation for the banding profiles after RE digestion in some cases (Gosálvez et al. 1986; Marchi and Mezzanotte 1990). Burkholder and Weaver (1977), analyzing the interactions between DNA and proteins in the condensed chromatin of rats and humans, observed a differential sensitivity to enzymatic digestion in some chromosomal regions according to differences in the DNA-attached proteins once they would protect them from enzymatic digestion. However, the relationship of this interaction to chromosomal banding differentiation has not been fully understood yet. In addition, conformational changes in chromosomal structure putatively account for RE digestion patterns in human chromosomes for instance (Mezzanotte et al. 1985).

**Figure 6.**
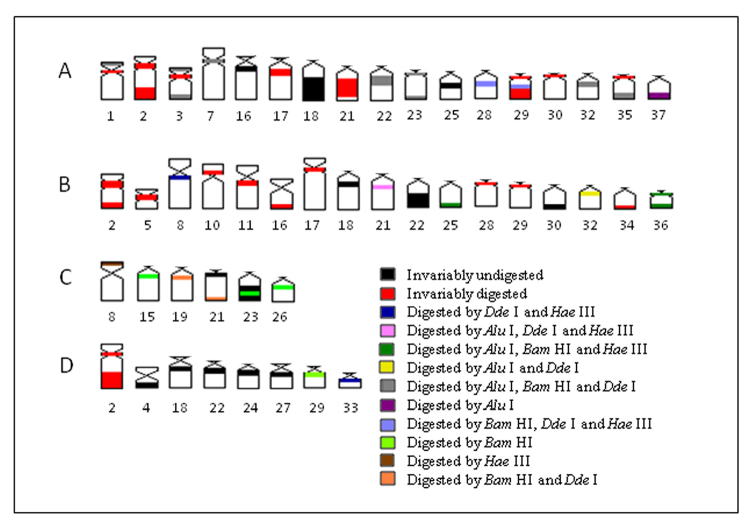
** A–D** Schematic ideogram of chromosomal pairs from populations **A, B, C** and **D** of *Hypostomu*s prope *unae*, showing the combined banding pattern after digestion using *Alu* I, *Bam* HI, *Hae* III and *Dde* I.

In the present work, the application of endonuclease treatments revealed a remarkable heterogeneity within heterochromatin among populations of *Hypostomus* prope *unae*, comprising either distinct or similar chromosomes, and even between heterochromatic segments. Based on these results, it was possible to identify inter- and intra-population (dis)similarities (Fig. 6). Most likely, the tested enzymes cleaved and removed DNA from both euchromatin and heterochromatin as demonstrated by some less stained chromosomal regions. Therefore, the observed bands can be regarded as non-removed DNA portions lacking the RE target sequences.

The present data indicate that some heterochromatin regions in different chromosomes and/or populations share a similar composition, while others would present a unique composition. Thus, the banding pattern observed reflects directly the molecular nature of heterochromatin regions (Sánchez et al. 1991), although a differential access to target sequences by the RE might be present as well.

Such remarkable heterogeneous banding pattern shows that the populations of *Hypostomus* prope *unae* bear several heterochromatin families composed of distinct specific types of highly repetitive DNA. A similar finding was reported in the salmonids *Salmo salar* Linnaeus, 1758(Albuín et al. 1994) and *Salmo trutta* Linnaeus, 1758 (Sánchez et al. 1991), in which RE digestion resulted in differential heterochromatin digestion in specific chromosomal regions.

According to Schweizer and Loidl (1987), a non-random arrangement of chromosomes during interphase might favor the linkage between certain chromosomal regions and further heterochromatin dispersal to equilocal sites from one chromosome to another, as previously proposed for the distribution of interstitial heterochromatin in other *Hypostomus* species (Artoni and Bertollo 1999). It seems plausible to infer that those heterochromatin segments sharing a similar composition would have a common origin and have been dispersed to similar chromosomal regions of *Hypostomus* prope *unae*. Through their karyoevolutionary history, these segments could have been amplified or accumulated by unequal exchanges, transpositions and/or regional duplications as similarly hypothesized for the marine fish *Centropyge aurantonotus* Burgess, 1974 (Affonso and Galetti Jr. 2005). Consequently, the chromosomal divergence among the studied populations have possibly been related to rearrangements in the heterochromatin organization and fixed either by genetic drift or by natural selection if some adaptive role is assumed.

Although inter-population differences were detected by both C-banding and RE digestion, some heterochromatin regions remained resistant to enzymatic digestion among populations, mainly in population D, revealing a higher differentiation in the DNA composition and/or heterochromatin organization in the latter. This population is also more divergent than the others because of its high frequency of interstitial C bands instead of terminal ones (Figs 5, 6).

Differences in heterochromatin patterns have been commonly reported in Neotropical fishes, including species from northeastern coastal basins (Jacobina et al. 2009). However, evolutionary mechanisms of heterochromatin differentiation among fish populations are usually related to polymorphic conditions being rarely detected within a single basin (Molina et al. 2008). Thus, the present results indicate that gene flow among *Hypostomus* prope *unae* along Contas river basin is absent, favoring the fixation of divergent heterochromatin patterns.

It should be pointed out that the nucleolar organizer regions (2^nd^ pair) was digested by all tested enzymes independently on the population, demonstrating that the distinct target sequences are “concertedly” interspersed along this region, even when NOR-associated heterochromatin was not detected, as observed in population C. Such behavior differs from the pattern observed by Sanches et al. (1990) that reported a differential NOR digestion indicative of a high amount of target sequences for *Dde* I and *Hae* III but a moderate number of restriction sites for *Alu* I.

Moreover, heteromorphic segments were observed between some chromosomal pairs in populations A (pairs 18, 21 and 37) and B (pair 22). Nonetheless, only the 21^st^ pair in population A presented the target sequences for the selected RE, while the other heteromorphic segments proved to be resistant to their digestion activity.

Reports about restriction enzymes in Neotropical fish cytogenetics are scarce what hinders a detailed comparative analysis. However, this approach seems to be highly informative for species characterized by large amounts of heterochromatin as that presently studied, being able to reveal several genomic particularities. Moreover, repetitive DNA sequences might provide efficient chromosomal markers useful for evolutionary studies, identification of chromosomal rearrangements and sex differentiation (Ferreira and Martins 2008).

As commonly reported in fishes of the genus *Hypostomus* (e.g., Milhomem et al. 2010), the present cytogenetic analyses were able to differentiate the four studied populations of *Hypostomus* prope *unae*, thereby reinforcing their evolutionary divergence along Contas river basin and their cryptic species diversity.
